# Cyberknife Radiosurgery for the Treatment of Head and Neck Cancer: A Systematic Review

**DOI:** 10.1055/s-0041-1736330

**Published:** 2021-12-10

**Authors:** Cristalle Soman, Shahad Ramzi Mohammed Alghamdi, Faisal Nahar M. Alazemi, Abdulaziz Ahmad Abdullah Alghamdi

**Affiliations:** 1 Department of Oral Medicine and Maxillofacial Radiology, Riyadh Elm University, Riyadh, Saudi Arabia; 2 College of Dentistry, Riyadh Elm University, Riyadh, Saudi Arabia; 3 College of Dentistry, Prince Sattam Bin Abdulaziz University, Al-Kharj, Saudi Arabia

**Keywords:** cyberknife radiosurgery, oropharyngeal neoplasms, treatment outcomes of cyberknife, cyberknife radiotherapy, head and neck neoplasm, targeted radiation therapy, stereotactic radiotherapy, robotic radiosurgery

## Abstract

Cyberknife radiosurgery is a frameless stereotactic robotic radiosurgery which has shown to deliver better treatment outcomes in the treatment of advanced head and neck (H&N) carcinomas, especially in previously irradiated and recurrent cases. The aim of the study was to perform a systematic review of the available data on the outcomes of Cyberknife radiosurgery for treatment of head and neck cancer and to evaluate its collective outcomes. This systematic review was registered with the university with the registration no. FRP/2019/63 and was approved by the Institutional Review Board (RC/IRB/2019/132). Literature search was performed in the following: PubMed, Science direct, SciELO, MyScienceWork, Microsoft Academ EMBASE, Directory of Open Access Journals, and Cochrane databases with the keywords “Cyberknife,” “oral cancer,” “oropharyngeal cancer,” and “head and neck cancer” and data was extracted according to the Preferred Reporting Items for Systematic Reviews and Meta-Analyses (PRISMA) guidelines. The records identified were 147 manuscripts. Excluded articles included 5 duplicate articles, 33 abstracts, 101 full text articles due to being off-topic, case reports, review, non-English, 1 survey, and 2 other articles containing data extracted from a main study which was already included. A total of 5 articles were evaluated for qualitative synthesis. The mean dose of Cyberknife radiosurgery delivered for previously irradiated recurrent H&N carcinoma patients was 34.57 Gy, with a mean sample size of 5 studied during the period of 2000 to 2016. The available evidence from the systematic review indicates that Cyberknife can be an efficacious treatment option for recurrent previously irradiated H&N carcinoma, especially for nonresectable tumors. There is paucity of homogenous data and studies in this arena; hence, meta-analysis could not be performed. Further standardized studies are essential, especially where the treatment of H&N carcinoma is considered.

## Introduction

The concept of Cyberknife radiosurgery was invented by a Stanford neurosurgeon, Dr. John Adler, and came into effective practice by 1900. Cyberknife is a stereotactic radiosurgical unit capable of irradiating the tissues using ionizing radiation. The ability of beam shaping using multilamellar collimators and three-dimensional conformation of the target tissue with submillimeter accuracy make this radiosurgical system stand out from the other radiosurgical units which has accuracy in millimeters. This highly precise dose delivery enables minimal damage to the surrounding structures. 1 2

Cyberknife is used for the treatment of benign and malignant lesions as well as intracranial and extracranial lesions, including whole body radiosurgery. 1 Cyberknife is most apt in the treatment of previously irradiated or recurrent or residual tumors and also gives radiobiological advantage and palliative care to patients with poor prognosis and performance status. 1 3

Cyberknife has also been widely used in the treatment of head and neck (H&N) cancers and lesions. However, no systematic reviews have been conducted to determine its efficiency. Hence, this systematic review was conducted to provide valuable information on the treatment efficacy and analyze the outcomes of the treatment with Cyberknife.

## Methods

The research proposal was registered with the university with the registration no. FRP/2019/63 and was approved by the Institutional Review Board (RC/IRB/2019/132). A meticulous literature search was made in the following major search engines: PubMed, Science direct, SciELO, MyScienceWork, Microsoft_Academ EMBASE, Directory of Open Access Journals, and Cochrane databases with the keywords “Cyberknife,” “oral cancer,” or “oropharyngeal cancer,” and “head and neck cancer.” This study was conducted in accordance with the Preferred Reporting Items for Systematic Reviews and Meta-Analyses (PRISMA) guidelines. 4 A total of 147 manuscripts were found and reviewed. Surveys, case reports, reviews, abstracts, non-English, and off-topic articles were excluded. Studies with full-text articles published in English were included in the analysis, assessed systematically, and outcomes were evaluated. Data was extracted for patient demographics, number of patients, tumor size, median dose, follow-up duration, overall survival rates, and toxicities.

Gross tumor volume (GTV) was defined as the volume of visible tumor, as evaluated from imaging studies. 5 Planned tumor volume (PTV) was obtained by adding 1 to 3 mm to the GTV, depending on proximity to surrounding structures, tumor geometry, and prior treatment outcomes. 6 Tumor response evaluations were based on response evaluation criteria in solid tumors (RECIST) as follows 7 :

- Complete response (CR) was defined as disappearance of all target lesions.
- Partial response (PR) was defined as 30% decrease in the sum of the longest diameter of target lesions.
- Progressive disease (PD) represented 20% or greater increase in the sum of the longest diameter of target lesions.
- Stable disease (SD) represented targets where there is neither sufficient shrinkage to be termed as PR nor sufficient increase to qualify it as PD.

Overall survival (OS) represents the period from the start of reirradiation radiotherapy to the date of demise from any cause or till the last follow-up. 6

## Results

**Fig. 1:**
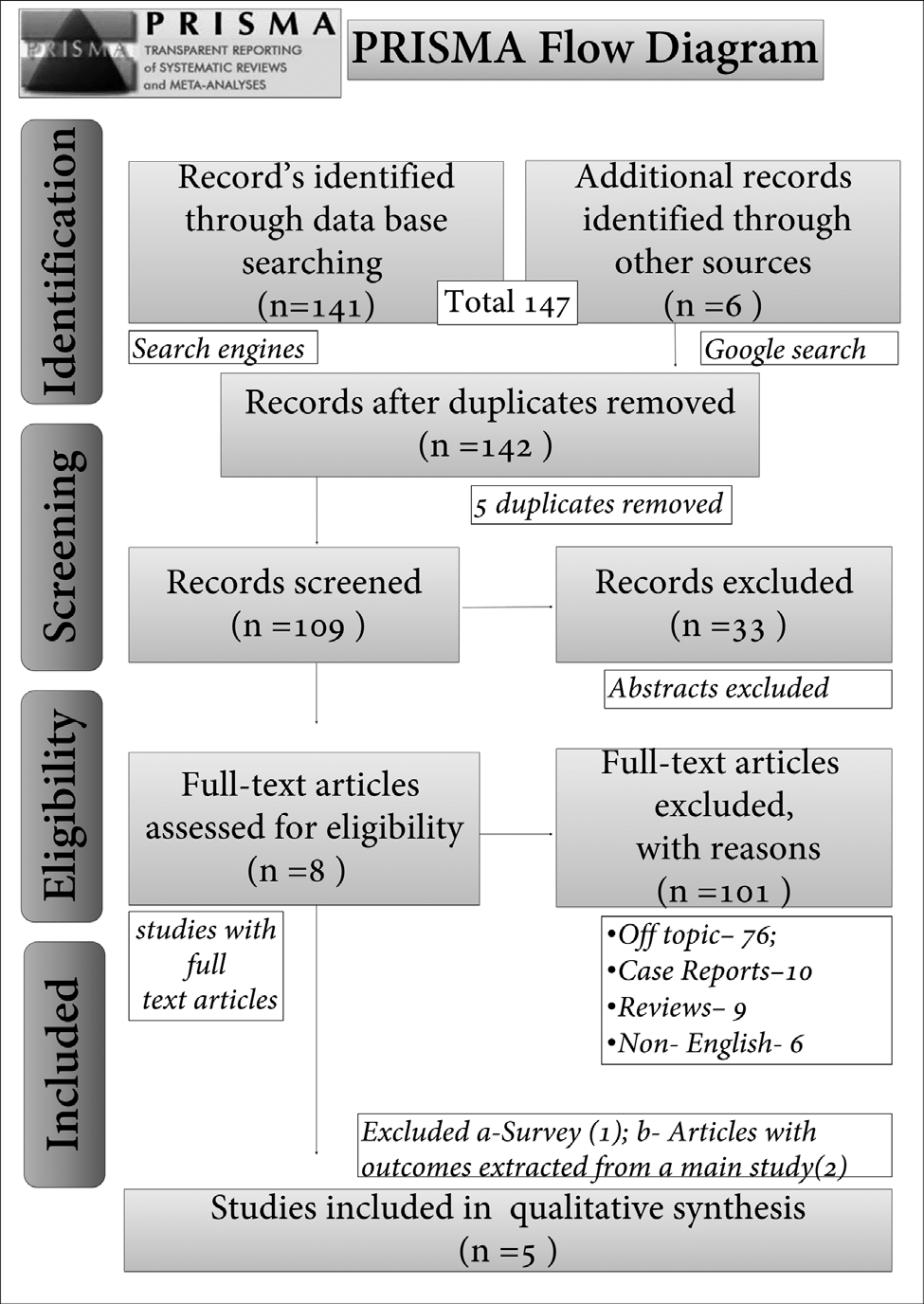
Preferred Reporting Items for Systematic Reviews and Meta-Analyses (PRISMA) flowchart of included studies.

From identification to eligibility phase, all authors (S.R.M.A., F.N.M.A., A.A.A.A.) individually reviewed and compiled data with the guidance and supervision of C.S. Database search contributed to 141 articles and a Google search for additional data retrieved 6 articles, with a total number of 147 manuscripts in the identification phase. Five duplicates were removed, and 33 abstracts were removed during screening. For assessment of eligibility, 101 articles were excluded due to the following reasons: off topic—76, case reports—10, reviews—9, non-English articles—6. Further, three articles were excluded: 1 survey and 2 articles which projected outcomes from one main study, which was already included for final phase of systemic analysis including 5 articles ( Fig. 1 ).

After careful scrutiny of the published data, C.S. evaluated the articles to be included in the qualitative synthesis, with a total of 5 articles included for data extraction on the outcomes of Cyberknife radiotherapy for (H&N) cancer treatment, comprising 275 patients studied during 2000 to 2016. Data was extracted and cross-evaluated by all the authors. Details of the included studies with their sample sizes and demographics are presented in Table 1 . The mean sample size was 55 (range, 22–107), with a gender distribution of 196 (71.27%) males and 79 (28.73%) females. All the participants in the studies had undergone previous radiotherapy, except in one study group in which patients underwent previous surgery/chemotherapy/radiotherapy. The median age range of all the studies were within 61 to 67 years, with a minimum age of 35 years and maximum of 91 years. Table 1 also presents data on history of prior surgery/chemotherapy/radiotherapy or a combination of the aforementioned treatments. Previous radiotherapy dosage ranged from a minimum of 30 Gy to a maximum of 170.7 Gy. Details on the primary site distribution, median interval between initial or previous irradiation and Cyberknife radiotherapy has been presented in Table 1 . This table also provides information on the risk of bias involved in each study analyzed, using Cochrane Collaboration's tool for assessing risk of bias 8 (adapted from Higgins and Altman 9 ).

**Table 1:** Details of published studies

Sl. no.	Study details authors	Institution; study years	Study design	Pretreatment diagnosis	Patients, *n*	Gender, *n* (%)	Median age, in years (range)	Previous surgery/chemotherapy/reirradiation, *n* (%)	Median dose of previous radiotherapy, Gy (range)	Primary site	Median interval between initial or previous irradiation and Ck SBRT in months, ( *n* )	Risk of bias
1	Stanisce et al 6	Cooper University Hospital; (2011–2016)	RET-Ck	Previously irradiated recurrent H&N carcinoma	25	19M:6F (76:24%)	64 (43–85)	S-14 (56%) C-20 (80%) R-3 (12%)	70 (30–110)	To:9 (36%) NP:3 (12%) PTG: 3 (12%) HP: 2 (8%) OP: 2 (8%) TSL: 2 (8%) EG: 1 (4%) LNX: 1 (4%) MS: 1 (4%) LCM: 1 (4%)	=13, (11)	Selection bias Attrition bias Imprecision bias (low risk) (small sample)
2	Yamazaki et al 5	Soseikai General, Fujimoto Hayasuzu, Okayama Kyokuto, Osaka University Hospitals and Hyogo Ion Beam Medical Center (2000–2010)	RET cohort Ck vs. CP	Previously irradiated recurrent H&N carcinoma	25	21M:4F (84:16%)	61 (42–88)	S-13 (52%) NS-12 (48%)	60 (40–116)	Oral: 4 (9%) NP: 3 (12%) SG: 0 (0%) HP-OP: 9 (20%) N-PNS: 9 (20%) LCM: 0 (0%) Other: 22 (88%)	≤ 30, 19 ≥ 30, 6	Selection bias; Imprecision bias (low risk) (small sample)
3	Yamazaki et al 34	Soseikai General, Fujimoto Hayasuzu, Okayama Kyokuto, and Osaka University Hospitals (Japan) (2000–2010)	RET; Multi-institutional review Ck	Previously irradiated recurrent H&N carcinoma	107	78M:29F (73:27%)	63 (35–88)	S-47 (43) NS-60 (55)	60 (40–116)	NP: 41 (38) OP21 (19) HP11 (10) Oral 14 (13) N-PNS: 20 (18)	≤ 30 = 71 > 30 = 36	Selection bias, physician-based bias may exist.
4	Rwigema et al 35	University of Pittsburgh Cancer Institute (January 2003 and October 2008)	RET cohort Ck-IMRT	Previously irradiated recurrent H and Neck Carcinoma	96	70M:26 F	67 (39- 88)	68.4 (32–170).	68.4 (32–170.7)	NP: 7 (7.) PTG: 4 (4.2) HP and neck: 23 (24) OP: 8 (8.3) PNS: 5 (5.2) Oral: 26 (27.1) Base of the skull:5 (5.2) Orbit: 2(2.1) Outer ear: 2 (2.1) Other: 1 (1)	No data available	Physician-based bias may exist. Selection bias
5	Kawaguchi et al 36	Yokohama Ck Center, Japan (2006–2007)	PRO	Advanced, recurrent head and neck cancer with previous surgery/chemotherapy/irradiation	22	8M:14F (64:36%)	67 (42–91)	S-8 (36%) S + C + R-13 (59%) R-1 (5%)	40–65; no data on median value	Data not available	No data available	Selection bias Precision bias Missing data (low risk) Imprecision bias (low risk) (small sample)

Abbreviations: C, chemotherapy; Ck, Cyberknife; CP, charged particle; EG, epiglottis; HP, hypopharynx; H&N, head and neck; IMRT, image-modulated radiotherapy; LCM, lacrimal gland; LNX, larynx; MS, maxillary sinus; NP, nasopharynx; N-PNS, nasal-paranasal sinuses; OP, oropharynx; PRO, prospective; PTG, parotid gland; R, radiotherapy; RET, retrospective; S, surgery; To, tongue; TSL, tonsils.

Table 2 details the outcomes of the Cyberknife treatment for H&N cancer. Among the five studies evaluated and analyzed for eligibility, only three studies were included for quantitative outcome assessment ( Table 3 ). Two studies were excluded due to their study design, absence of coherence with the tumor size volume calculated, and nondetailed description of tumor control. After sorting of the data on outcomes, three studies were analyzed for eligibility for quantitative synthesis. However, these three studies lacked homogenous study design parameters such as lack of concurrent chemotherapy and factors for outcome assessment such as nonhomogenous evaluation of tumor control, median survival rate, and mean follow-up. Due to the aforementioned factors, meta-analysis for the studies could not be performed. The mean survival rate after Cyberknife radiotherapy in the previously irradiated H&N carcinoma patients was 10.9 months. Eleven patients experienced carotid blowout syndrome (CBOS). Grade 1 and grade 2 toxicities were reported to be maximum. The descending order of toxicities reported is as follows: grade 1 + grade 2 > grade 3 > grade 5 > grade 4. The patients with ulceration at the time of presentation for Cyberknife treatment, tumor volume size, and presence of metastasis were found to influence the tumor response in the patients undergoing Cyberknife treatment. No cases of distant metastatic progression was reported among 275 patients.

**Table 2:** Outcomes of the Ck treatment for H&N cancer

Sl.no.	Study	Patients, *n*	Pretreatment tumor size/volume	Median dose (Gy)	Dosing strategy; Fractionated sessions	Concurrent chemotherapy	Tumor control in patients *n* (%)	Overall survival rate at 1 year and 2-year intervals	Median survival rate in months (range)	Any other factors affecting tumor response/OS	Mean follow-up range (in months)	Major complications, *n*
1	Stanisce et al 6	25	PTV = 31.75 (5.5–121.9) (+1–3 mm)	40 (24–44)	5 (3–5)	11	PD = 0 LF-8 (32%) LRF (28%) DMP-0	32% (1 year) 16% (2 year)	7.5 (1.5 -47)	OS without metastasis 8.3 months OS With metastasis 2 months	1–3 months; every 3 months (Mean and range not available *)*	Toxicity a Grade 1–2 = 10 Grade 3 = 1 (palliative) Grade 4/5 = 0 CBOS = 0
2	Yamazaki et al 5	25	PTV = 4 (16%) in ≤ 40 cm ^3^ PTV = 21 (84%) in > 40cm ^3^ (PTV = GTV+ 0–3 mm)	32 (25–39)	5 (3–8)	0	LC 63.8%	All patients 36.3% (1 year)	6.6 (Range not available)	None	8 (0.4–54.5)	Toxicity b Grade 0–2 = 19 Grade 3 = 4 Grade 4 = 0 Grade 5 = 2 CBOS = 0
3	Yamazaki et al 34	107	PTV = 28.4 (1–339)	30 (15–39)	5 (3–8)	0	CR + PR-54% c SD = 36.44% PD-8.4% LF-0 LRF-35 (33%) DMP-0 LRC 64% (2 years)	55 (1 year) and 35% (2 year)	14.4 (10.8–19.4)	Ulceration +lower response rate (28%: 1 CR + 6 PR = 7/25) the ulceration (−) group 63%: 22 CR + 29 PR = 51/81) ( *p* = 0.0045).	15 (10–122).	Toxicity b Grade 1–2 = 0 Grade 3 = 13 Grade 4 = 2 Grade 5 = 9 CBOS = 11
4	Rwigema et al 35	96	GTV 24.3 (2.5–162) No details on PTV	35 (15–50)	8 (4–10)	58	Tumor volume ≤ 25: 72.6% (1 year) and 67.4% (2 year), > 25 cm ^3^ ) at 48.2% (1 year) 18.6% (2 year), ( *p* = 0.007 c	58.9% (1 year) 28.4% (2 years)	15	Tumor volume	14 (2–39).	Grade 1 = 37.5%, Grade 2 = 17.7%, and Grade 3 = 5.2%.
5	Kawaguchi et al 36	22	PTV = GTV = 24.5 (3.4 - 74.4)	33.7 (20–42)	2–5	22 d	CR-10 (45.5%) c PR-1 (7.1%) SD-1 (7.1%) PD-10 (45.45%)	78.8% at 2 years	No data available	No data available	24 (4–39)	Toxicity b Grade 1–2= 28 Grade 3 = 5 Grade 4 = 0 Grade 5 = 0 CBOS = 0

Abbreviations: CBOS, carotid blowout syndrome; Ck, Cyberknife; CR, complete response; DMP, distant metastatic progression; GTV, gross tumor volume; H&N, head and neck; LF, local failure; LRC, locoregional control; LRF, locoregional failure; OS, overall survival; PD, progressive Disease; PR, partial response; PTV, planned tumor volume; SD, stable disease.

a Toxicity grades were based upon various versions of the Common Terminology Criteria for Adverse Events (CTCAE).

b Toxicity was evaluated using the National Cancer Institute Common Toxicity Criteria scale version 3.0.

c Treatment outcome was assessed based on the Response Evaluation Criteria in Solid Tumors (RECIST).

d Yes low dose.

**Table 3:** Evaluation for quantitative analysis on outcomes of the Ck treatment for H&N cancer

Sl. no.	Study	Patients, *n*	Pretreatment tumor size/volume	Median dose (Gy)	Dosing strategy; fractionated sessions	Concurrent chemotherapy	Tumor control in patients *n* (%)	Overall survival rate at 1 year and 2-year intervals	Median survival rate in months (range)	Any other factors affecting tumor response/OS	Mean follow-up range (in months)	Major complications, *n*
1	Stanisce et al 6	25	PTV = 31.75 (5.5–121.9) (+1–3 mm)	40 (24–44)	5 (3–5)	11	PD = 0 LF-8 (32%) LRF (28%) DMP-0	32% (1 year) 16% (2 years)	7.5 (1.5–47)	OS without metastasis 8.3 months OS with metastasis 2 months	1–3 months; every 3 months (mean and range not available)	Toxicity **a** Grade 1–2 = 10 Grade 3 = 1 (palliative) Grade 4 = 0 Grade 5 = 0 CBOS= 0
2	Yamazaki et al 5	107	PTV = 28.4 (1–339)	30 (15–39)	5 (3–8)	0	CR + PR 54% c SD = 36.44% PD-8.4% LF-0 LRF-35 (33%) DMP-0 LRC 64% (2 years)	55 (1 year) and 35% (2 years)	14.4 (10.8–19.4)	Ulceration + lower response rate (28%: 1 CR + 6 PR = 7/25) the ulceration (−) group 63%: 22 CR + 29 PR = 51/81) ( *p* = 0.0045)	15 (10–122).	Toxicity b Grade 1–2 = 0 Grade 3 = 13 Grade 4 = 2 Grade 5 = 9 CBOS = 11
3	Kawaguchi. et al 34	22	PTV = GTV = 24.5 (3.4–74.4)	33.7(20–42)	2–5	22 d	CR-10 (45.5%) c PR-1 (7.1%) SD-1 (7.1%) PD-10 (45.45%)	78.8% at 2 years	No data available	No data available	24 (4–39)	Toxicity b Grade 1–2 = 28 Grade 3 = 5 Grade 4 = 0 Grade 5 = 0 CBOS = 0

Abbreviations: CBOS, Carotid blowout syndrome; CR, complete response; DMP, distant metastatic progression; GTV, gross tumor volume; LF, local failure; LRC, locoregional control; LRF, locoregional failure; OS, overall survival; PD, progressive disease; PR, partial response; PTV, planned tumor volume; SD, stable disease.

a Toxicity grades were based upon various versions of the Common Terminology Criteria for Adverse Events (CTCAE).

b Toxicity was evaluated using the National Cancer Institute Common Toxicity Criteria scale version 3.0.

c Treatment outcome was assessed based on the Response Evaluation Criteria in Solid Tumors (RECIST).

d Yes low dose.

## Discussion

In this study, we evaluated the treatment efficacy of Cyberknife for the treatment of H&N cancers on 275 patients between the years 2000 to 2016. The outcomes assessment for CK treatment of H&N cancer was assessed on locoregional tumor control, overall survival rate at 1 year and 2 years, median survival rate, mean follow-up period, and development of major complications during the period of follow-up. Cyberknife treatment was found to have a good loco regional tumor control, 10 11 12 13 14 15 with absence of distant metastatic progression in majority of the cases of previously irradiated H&N cancers with or without concurrent chemotherapy. The overall survival rate was found to decrease from first year to second year of follow-up period, except for a fairly good survival rate in the study conducted by Kawaguchi et al which is 78.8% at 2 years. This increase in survival rate in the aforementioned study can be due to the low sample size in the study. The mean survival rate at 2 years was 43.33%. The overall survival rate using Cyberknife was found to be more than other stereotactic body radiotherapy (SBRT) methods like image-modulated radiotherapy (IMRT). 10 11 12 13 14 15 Mean follow-up range was 19.5 from the two studies by Yamazaki et al and Kawaguchi et al.

Radiotherapy for the management of cancers were used as one of the methods of primary management in the early 20th century due to limitations of anesthesia and surgical outcomes. However, prior to 1948, radiotherapy administration was limited to radium implants and orthovoltage radiation with insufficient depth of penetration into the tissues and increase in severity of skin damage. 16 Toward the middle of the 20th century, surgical management became the primary modality with or without concurrent radiotherapy due to several factors like advancement in perioperative and postoperative care and early radiotherapy treatment failures. In the 1950s, the advent of telecobalt units and linear accelerator radiotherapy and research and advancements in radiotherapy improved treatment outcomes as well as reduction in toxicities following which radiotherapy gained a prime importance in management of cancers. 16 17 18 19 However, it was after the second half of the 20th century, preference was diverted to functional outcomes and advent of chemotherapy as an adjuvant or concurrent method of treatment was evolved. 20 The overall survival rates for patient receiving radiotherapy increased thereafter in the following years. 19 21 Radiotherapy is now used as a primary treatment strategy for oropharyngeal and hypopharyngeal carcinoma management due to the rate of tumor control and lower morbidity compared with surgical therapy. 22 23 The evolution of SBRT changed the phase of radiotherapy by offering a tumor specific target doses, sparing the vital structures with minimal morbidity. 24 25 26 27 28 29 Sterotactic radiotherapy, coined in the 1950s by Dr. Lars Lekshell, is a method of radiotherapy which involves fractionated dose delivery with highly conformal tissue exposure to radiation dose. 16 30 SBRT included IMRT, Gammaknife, and Cyberknife. 31

Gammaknife was invented in 1972, and it is currently one of the prominent radiotherapy delivery units. However, it requires skeletal fixation, which can cause pain, thereby restricting flexible and highly precise conformal radiotherapy. 31 It was Sir Dr. John Adler who invented the Cyberknife system which offers submillimeter accuracy with highly conformal radiotherapy, three-dimensional accuracy, and preservation of proximal vital structures. 1 32 33

Cyberknife radiotherapy can be used for complex tumors such as around the spine in which surgical outcomes may be undesirable, cases of recurrent tumors with proximity to vital structures, as a concurrent or adjuvant for remaining inaccessible tumor after surgery, as a palliative therapy, and also in cases of previously irradiated cases of H&N cancers with or without concurrent radiotherapy. 6 7 8 31 32 34 35 36 There has been only a single published study on Cyberknife meta-analysis related to the treatment outcomes of vestibular schwannomas, 33 and it was concluded that Cyberknife was found to be an efficient radiotherapeutic technique with good tumor control, preserving vitality of proximal structures with comparatively less toxicities. 33

In the present systematic review, we also found results similar to the previously quoted 2017 study by Mahboubi et al for vestibular schwannomas using Cyberknife. All the patients analyzed in this study were previously irradiated/treated H&N carcinoma patients with recurrence at the time of Cyberknife radiotherapy. The mean survival rate was 10.9 months after Cyberknife radiotherapy. Out of 275 patients, 11 patients experienced CBOS. However, it was not mentioned that whether the interval between previous radiotherapy and Cyberknife treatment in these 11 patients were less than 30 months or more than 30 months. CBOS is a life-threatening conditions seen in patients with H&N cancer. 37 Risk factors for development of CBOS in H&N cancer patients include radiotherapy, chemotherapy, chemoradiotherapy, surgery, fungating tumors, infections. Advanced tumor staging and local recurrence along with open surgical procedures also increase the risk of CBOD-related death. 37 38 39 40

Recent data from the International Agency for Research on Cancer (IARC) based on the 2018 report on lip and oral cavity cancers and associated death from 185 countries imparts light on the newly diagnosed cancer cases per year and that it could be considered to carry a high proportion of H&N cancers. 41 42 A recent study conducted by Grafton-Clarke et al in 2019 highlights the paucity of data on the primary care of oral cancer cases and delay in diagnosis in primary care. 42 This delay could results in tumor progression and spread, especially into inaccessible sites, invading vital structures and leading to high-mortality rates. 41 43 44 Despite the accessibility of fractionated radiotherapy, there is evidence that the vital structures may get affected like the salivary glands, leading to xerostomia and thereby affecting the quality of life postradiotherapy. 45 In such situations, stereotactic radiosurgery like Cyberknife is an invaluable treatment option and plays a vital role in tissue preservation. In dentistry, this feature plays a vital role in improving the potential for dental rehabilitation, improving the function and at the same time offering better quality of life. 46

There were several precincts in this study. The outcomes reported in the published studies have heterogenous methodology and parameters of assessment hindering the meta-analysis in all variables. Low samples size in majority of these studies was also a limiting factor. Association of outcome variables to previous treatment methodologies and its interval were not available. Selection bias was found to be common in many of the studies. All the studies included were not retrospective cohort studies. The factors listed above pose an inherent limitation in drawing conclusions regarding Cyberknife treatment in H&N cancer patients. Nevertheless, evaluation of the available data suggests that Cyberknife treatment for H&N cancers offers patients significant control on distant metastatic progression with preservation of vitality of adjacent vital structures and less toxicity. This study is the first study to systematically review the treatment efficacy of Cyberknife radiotherapy on previously irradiated recurrent H&N cancer.

## Conclusion

The mean dose of Cyberknife radiosurgery delivered for previously irradiated Recurrent H&N carcinoma patients was 34.57 Gy, with a mean sample size of 51 studied during the period of 2000 to 2016. Cyberknife treatment limits the occurrence of distant metastatic progression, thereby increasing the overall survival rates. The available evidence from the systematic review indicates that Cyberknife can be an efficacious treatment option for recurrent previously irradiated H&N carcinoma, especially for nonresectable tumors. There is paucity of homogenous data and studies in this arena. Further standardized studies are essential, especially where the treatment of H&N carcinoma is considered.
